# A Unique Case Series of Endometrial Vascular Dystrophy: Rising Incidence and Association With Uterine Pathologies

**DOI:** 10.7759/cureus.97704

**Published:** 2025-11-24

**Authors:** Nanda Jambunath Bugude, Saraswati Ramesh, Tasmia Akhtar

**Affiliations:** 1 Department of Obstetrics and Gynaecology, Bangalore Endoscopic Surgery Training Institute and Research Centre, Bangalore, IND; 2 Department of Obstetrics and Gynaecology, AV Hospital, Bangalore Endoscopic Surgery Training Institute and Research Centre, Bangalore, IND

**Keywords:** endometrial vascular dystrophy, endometriosis, hysteroscopy, intrauterine adhesions, isthmocele

## Abstract

Endometrial vascular dystrophy (EVD) is a rare hysteroscopic finding characterized by abnormal, tortuous, and dilated endometrial vessels, most often associated with abnormal uterine bleeding (AUB). Despite its potential clinical impact, global literature on EVD remains scant. This case series describes three women diagnosed with EVD. Case 1 involved a 41-year-old multiparous woman presenting with heavy menstrual bleeding in the current cycle, which was earlier than usual; hysteroscopy revealed diffuse bluish, tortuous vessels coexisting with an isthmocele. Case 2 was a 50-year-old nulliparous woman with prolonged AUB and a history of MRI-guided high-intensity focused ultrasound ablation (MR-HIFU); hysteroscopy demonstrated diffuse EVD. Case 3 reported a 45-year-old woman on intermittent exogenous progesterone for a year, whose EVD distribution was sparse and observed alongside intrauterine adhesions. In each case, histopathology confirmed secretory endometrium with or without dilated glands. All the patients had symptom management. Notably, EVD changes coexisted with diverse endometrial pathological entities but were absent in isthmocele regions. A novel possible association of EVD with post-HIFU uterine changes was observed. This series illustrates the diagnostic value of hysteroscopy in identifying EVD as an under-recognized cause of AUB and highlights its possible coexistence with other endometrial pathologies. EVD appears benign and often self-limiting; however, its expanded recognition is essential, and further research is warranted to elucidate its long-term clinical significance, associations, and targeted management strategies.

## Introduction

Endometrial vascular dystrophy (EVD) represents a rare hysteroscopic finding characterised by the presence of tortuous, dilated, and occasionally thrombosed endometrial vessels [1]. The pathogenesis of EVD is unclear, with hypotheses suggesting hormonal influences, particularly by progesterone, may lead to exaggerated secretory changes in the endometrial microvasculature as observed in histopathological examinations [2]. Additionally, EVD has been described in association with acquired uterine anomalies such as isthmocele, although the nature and frequency of this association remain under investigation [3]. Despite sporadic case reports and small case series contributing to the literature, the spectrum of endometrial pathologies coexisting with EVD appears to be broader than previously recognised. Recent reports suggest that EVD may occur alongside intrauterine adhesions (IUAs), endometriosis, fibroid uterus, and in women with a history of non-invasive uterine procedures, such as high-intensity focused ultrasonography (HIFU) [3,4]. However, systematic characterization of these associations is limited. Furthermore, the spatial distribution of EVD-related vascular changes within the endometrial cavity, especially in relation to anatomical disruptions such as isthmocele, remains inadequately described.

## Case presentation

Case 1

**Figure 1:**
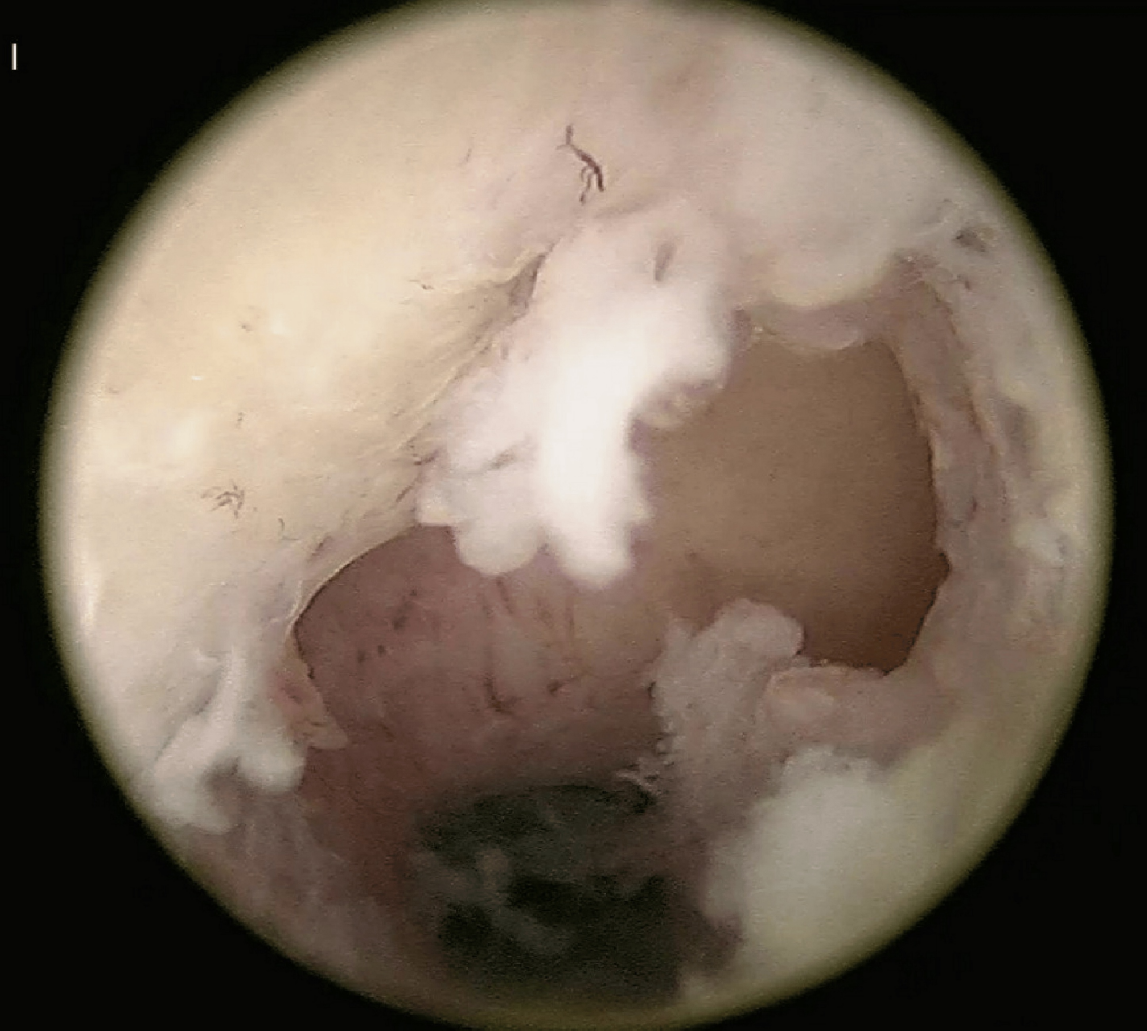
Isthmocele area devoid of endometrial vascular dystrophy Hysteroscopic image demonstrating the cesarean-section scar niche (isthmocele) appearing as a well-defined, translucent depression within the anterior uterine wall. The surrounding endometrium shows abnormal vessels, whereas the isthmocele region lacks the characteristic tortuous, bluish-hued vessels typical of endometrial vascular dystrophy (EVD).

A 41-year-old para 2L2 woman presented to our center with an 8-10-day history of heavy menstrual bleeding accompanied by passage of large clots. Her menstrual cycles had become irregular over the preceding cycle, occurring after 21 days instead of her customary 28-30 day interval. She reported progressive worsening of bleeding volume, and her current menstrual cycle bleeding was controlled with five days of progesterone hormone. The patient’s obstetric history included one term normal vaginal delivery and one lower-segment cesarean section performed 17 years prior. She denied intermenstrual spotting, postcoital bleeding, pelvic pain, or any systemic symptoms. There was no history of use of intrauterine devices, anticoagulants, or herbal supplements. Her basic laboratory investigations, including complete blood count, coagulation profile, and thyroid function tests, were normal. Transvaginal ultrasonography demonstrated a normally positioned, anteverted uterus measuring 8.2 × 5.1 × 4.7 cm, with a thickened endometrial stripe (12 mm) and right ovarian simple cyst measuring 3-4 cm in dimension. Given the history of heavy bleeding and ultrasound findings, diagnostic hysteroscopy was performed under general anesthesia. Hysteroscopic evaluation revealed intracervical adhesions, which were lysed at the outset of the procedure, and a regular uterine cavity with a well-defined isthmocele at the previous cesarean scar site (1) and diffuse, dilated, tortuous, bluish-hue vessels spanning the fundus and lateral walls (2), consistent with endometrial vascular dystrophy. Notably, the endometrial surface overlying the isthmocele appeared devoid of dystrophic vasculature (Figure 1). Regular blood investigations for all cases are summarised in Table 1.

**Table 1:** Summary of routine laboratory and vital parameters in three cases of endometrial vascular dystrophy (EVD) BP – Blood pressure; Hb – Hemoglobin; TLC – Total leukocyte count; PCV – Packed cell volume; MCV – Mean corpuscular volume; PT – Prothrombin time; aPTT – Activated partial thromboplastin time; INR – International normalized ratio; TSH – Thyroid-stimulating hormone; FT₄ – Free thyroxine; AST – Aspartate aminotransferase; ALT – Alanine aminotransferase; BUN – Blood urea nitrogen

Parameter	Units	Reference Range	Case 1	Case 2	Case 3
Age (years)	—	—	41	50	45
Pulse rate	beats/min	60–100	78	88	76
Blood pressure	mm Hg	90–140/60–90	118/74	126/82	114/70
Temperature	°C	36.0–37.2	36.7	36.8	36.6
Respiratory rate	Breaths/min	12–20	16	18	15
Hemoglobin (Hb)	g/dL	12–16	12.4	9.8	11.6
Total leukocyte count (TLC)	×10⁹/L	4.0–10.0	6.5	7.1	6.2
Platelet count	×10⁹/L	150–400	310	360	295
Packed cell volume (PCV)	%	36–46	38	32	36
Mean corpuscular volume (MCV)	fL	80–96	89	78	84
Prothrombin time (PT)	sec	11–14	12.3	12.8	12.1
Activated partial thromboplastin time (aPTT)	sec	25–35	29	30	28
International normalized ratio (INR)	—	0.8–1.2	1.0	1.1	1.0
Thyroid-stimulating hormone (TSH)	µIU/mL	0.4–4.2	2.1	3.8	2.4
Free thyroxine (FT₄)	ng/dL	0.8–1.8	1.2	1.0	1.3
Fasting blood glucose	mg/dL	70–110	92	88	95
Serum ferritin	ng/mL	15–150	45	12	35
Serum creatinine	mg/dL	0.6–1.1	0.8	0.9	0.7
Blood urea nitrogen (BUN)	mg/dL	7–20	12	14	11
Liver enzymes (AST/ALT)	U/L	10–40 / 10–40	26 / 28	22 / 25	24 / 27
Ultrasound uterus size	cm	—	8.2 × 5.1 × 4.7	194 × 72 × 152 with multiple fibroids	8.0 × 5.0 × 4.5
Endometrial thickness	mm	≤ 12 (proliferative)	12	18	10
Ovarian findings	—	—	Right simple cyst (3–4 cm)	Right hydrosalpinx + endometriotic cyst	Normal
Histopathology phase	—	—	Secretory	Secretory	Secretory
Special stain (Alcian blue)	—	—	Mucin positive	Mucin positive	Mucin positive

**Figure 2:**
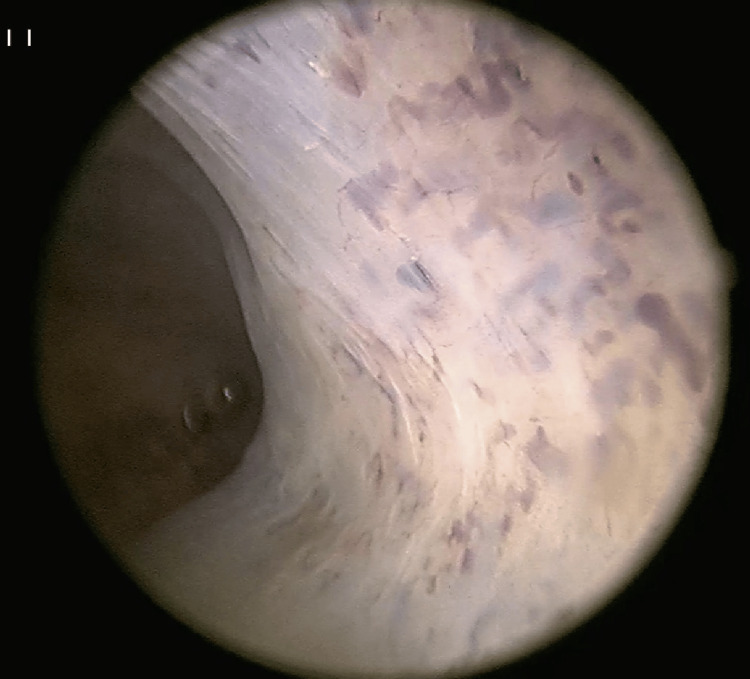
Hysteroscopic view of the left uterine wall demonstrating dilated, tortuous bluish vessels High-definition hysteroscopic visualization of the left lateral uterine wall revealing multiple dilated, serpentine vascular structures with a distinct bluish hue beneath the endometrial surface. These vessels represent the classical hysteroscopic hallmark of endometrial vascular dystrophy. The absence of active bleeding and the uniform distribution across the wall distinguish EVD from other vascular pathologies, such as arteriovenous malformations or retained products of conception. Image obtained using a 30° hysteroscope under saline distension.

**Figure 3:**
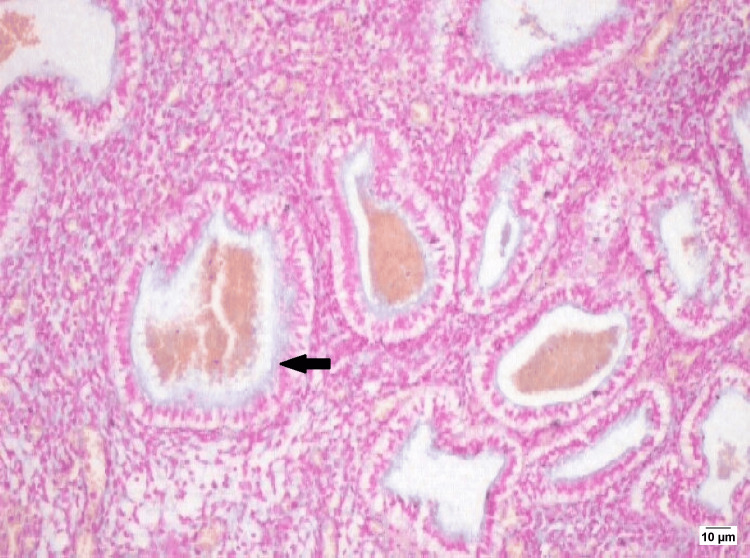
HPE-endometrial glands with mucin (blue colour) along the luminal surface The arrow mark shows endometrial glands with mucin with Alcian blue (100x)

Endometrial curettage was performed following hysteroscopic assessment, and tissue samples were sent for histopathological and special staining analyses. There was nil intervention for coexistent intra-operatively diagnosed isthmocele, which is an important pathology for AUB. Light microscopy revealed markedly dilated and tortuous endometrial glands containing entrapped erythrocytes, mimicking vascular channels. Alcian blue staining confirmed mucin within glandular lumina (3). Glands exhibited subnuclear and supranuclear vacuolation, indicative of the secretory phase (3). The exaggerated glandular appearance was attributed in part to recent exogenous progesterone exposure. Post procedure at six weeks and six months follow-up, she had complete resolution of heavy menstrual bleeding. This case emphasizes the importance of comprehensive hysteroscopic evaluation of the uterus with intraoperative diagnosis of EVD with isthmocele. The case underscores nil target-specific intervention performed towards pathologies such as EVD or isthmocele (Figures 2-3).

Case 2

**Figure 4:**
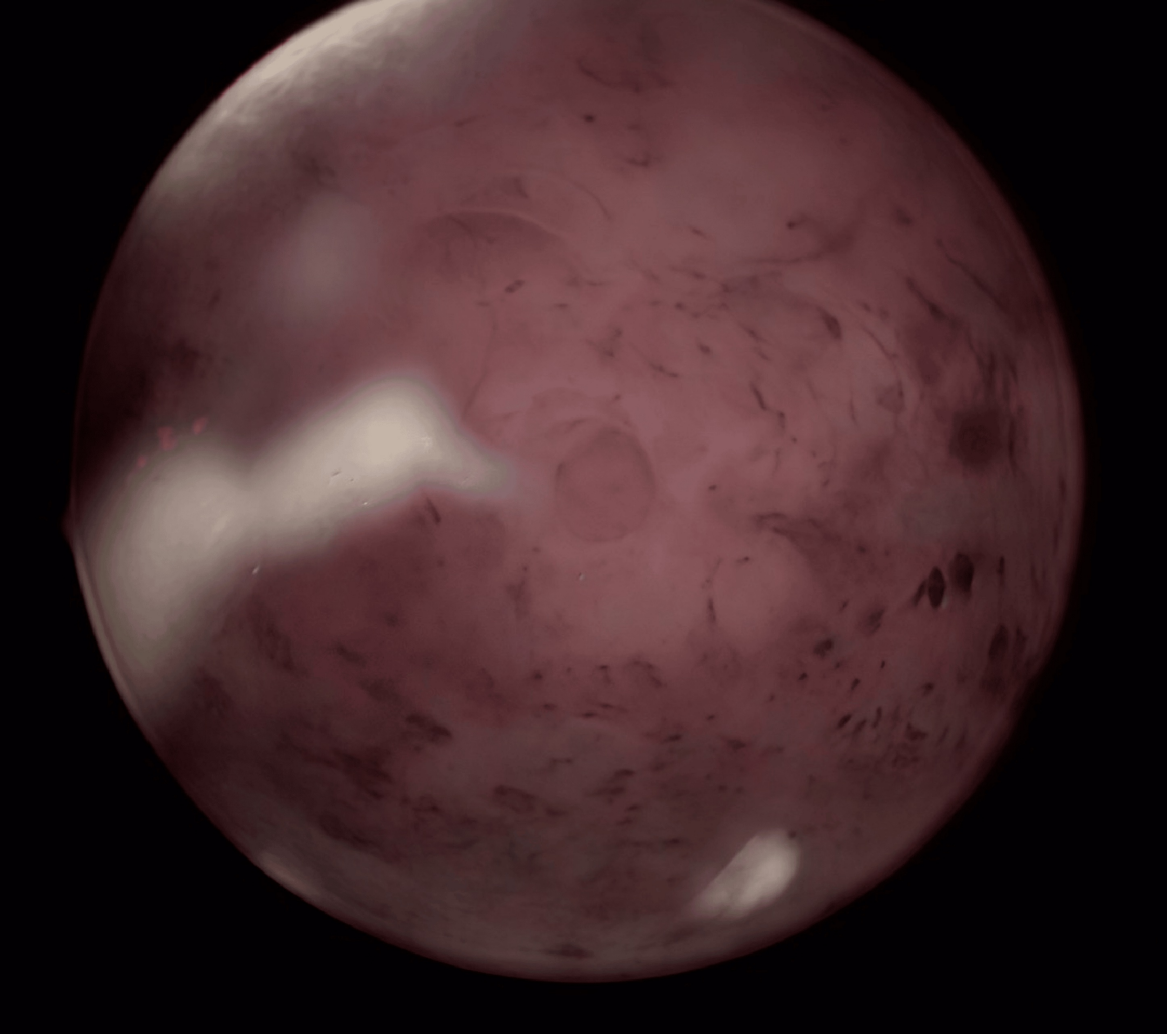
Uterine cavity exhibiting vascular dystrophy Ostial region hysteroscopic view of the uterine cavity showing widespread tortuous and dilated vascular channels distributed over the fundal and posterior walls. The image was captured during diagnostic hysteroscopy in a patient with a history of MR-guided high-intensity focused ultrasound (HIFU) treatment for fibroids. The diffuse vascular distortion and irregular endometrial texture demonstrate endometrial vascular dystrophy coexisting with leiomyomatous changes. The findings emphasize the role of prior uterine interventions and altered angiogenesis in EVD pathogenesis.

A 50-year-old nulliparous woman presented to us with a one and a half year history of menstrual irregularity and prolonged heavy bleeding during her current cycle. She reported continuous bleeding for 20 days with the passage of large clots and the need to change five to six sanitary pads per day. Initial medical management with oral tranexamic acid was ineffective, and she did not use any hormonal preparations to control her menstrual bleeding. She also gave a history of long-standing abdominal pain in her periumbilical region. Her past medical history was significant hypothyroidism managed on levothyroxine and iron deficiency anemia, for which she was on oral iron supplementation. At 37 years of age, she was diagnosed with multiple uterine fibroids detected on ultrasonography. At 42 years old, she underwent treatment for fibroids by MR-HIFU, achieving approximately 50-60 % necrosis of two dominant fibroids; no follow-up imaging was performed thereafter. At 50 years of age, she presented with persistent heavy menstrual bleeding unresponsive to medical tranexamic acid oral therapy and associated fatigue, but no pelvic pain, intermenstrual spotting, or systemic symptoms. On physical examination, the patient appeared pale. Abdominal palpation revealed a firm, irregular mass corresponding to a 20-week gravid-sized uterus. She also had a 2 cm reducible umbilical hernia. Her pelvic ultrasonography had a large fundal subserosal fibroid measuring 10.8 × 6.5 × 9.1 cm, a posterior wall intramural fibroid reduced to 2.2 × 1.9 cm, an endometrial thickness of 18 mm with internal cystic spaces, a right hydrosalpinx, a poorly visualized right ovary, and a 2-cm umbilical reducible hernial defect with bowel and omental content in the sac. Her magnetic resonance imaging (MRI) confirmed the large fundal fibroid and two smaller fibroids on the anterior and posterior uterine walls. A Pipelle aspiration, which was attempted due to thick endometrium in the clinic, was unsuccessful due to the bulky uterus and distorted cavity. Given the combination of symptomatic leiomyomas, thickened endometrium with cystic changes, and the patient not keen for uterine preservation, a shared decision was made in liaison with the multidisciplinary surgical team (MDT) to proceed with diagnostic hysteroscopy, endometrial sampling, and total laparoscopic hysterectomy with bilateral salpingectomy, along with concurrent umbilical hernia repair. Her hysteroscopy revealed diffuse dilated, tortuous vascular channels within the endometrial cavity - hallmarks of endometrial vascular dystrophy (Figure 4).

Endometrial sampling: hysteroscopy and endometrial curettage performed. With the help of surgical team expertise, an extensive adhesiolysis to release the bowel and right ovarian endometriotic cyst from the lateral pelvic walls, posterior uterus to the pouch of Douglas was done, and then they proceeded with a total laparoscopic hysterectomy with right salpingo-oophorectomy and left salpingectomy. As preoperatively planned, intraperitoneal onlay mesh (IPOM) repair of an umbilical hernia was performed by the general surgery team, with prophylactic left ureteric stent placement by urology to mitigate the risk of ureteral injury. The immediate postoperative course was uneventful. She was discharged on postoperative day two. At the three-week follow-up, the ureteric stent was removed without complication. Histopathological examination revealed.

Endometrium: Secretory phase endometrium with glands having red blood cells inside and alcian blue-positive mucin.

Myometrium: Hyaline degeneration within leiomyomas. Endometriotic cyst lining consistent with ovarian endometriosis.

At the six-month follow-up, the patient had significant improvement in her anemia and quality of life. This case highlights the value of comprehensive hysteroscopic evaluation of thickened endometrium and an enigmatic finding of rare endometrial vascular dystrophy in a secretory endometrium on HPE in association with endometriosis and fibroid uterus. This further raises the possibility of a correlation between the HIFU procedure and EVD occurrence. This case also elicited an optimal recovery despite both constructive (hernia repair) and destructive (hysterectomy) procedures performed concurrently, highlighting the benefits of combined laparoscopic procedures.

Case 3

**Figure 5:**
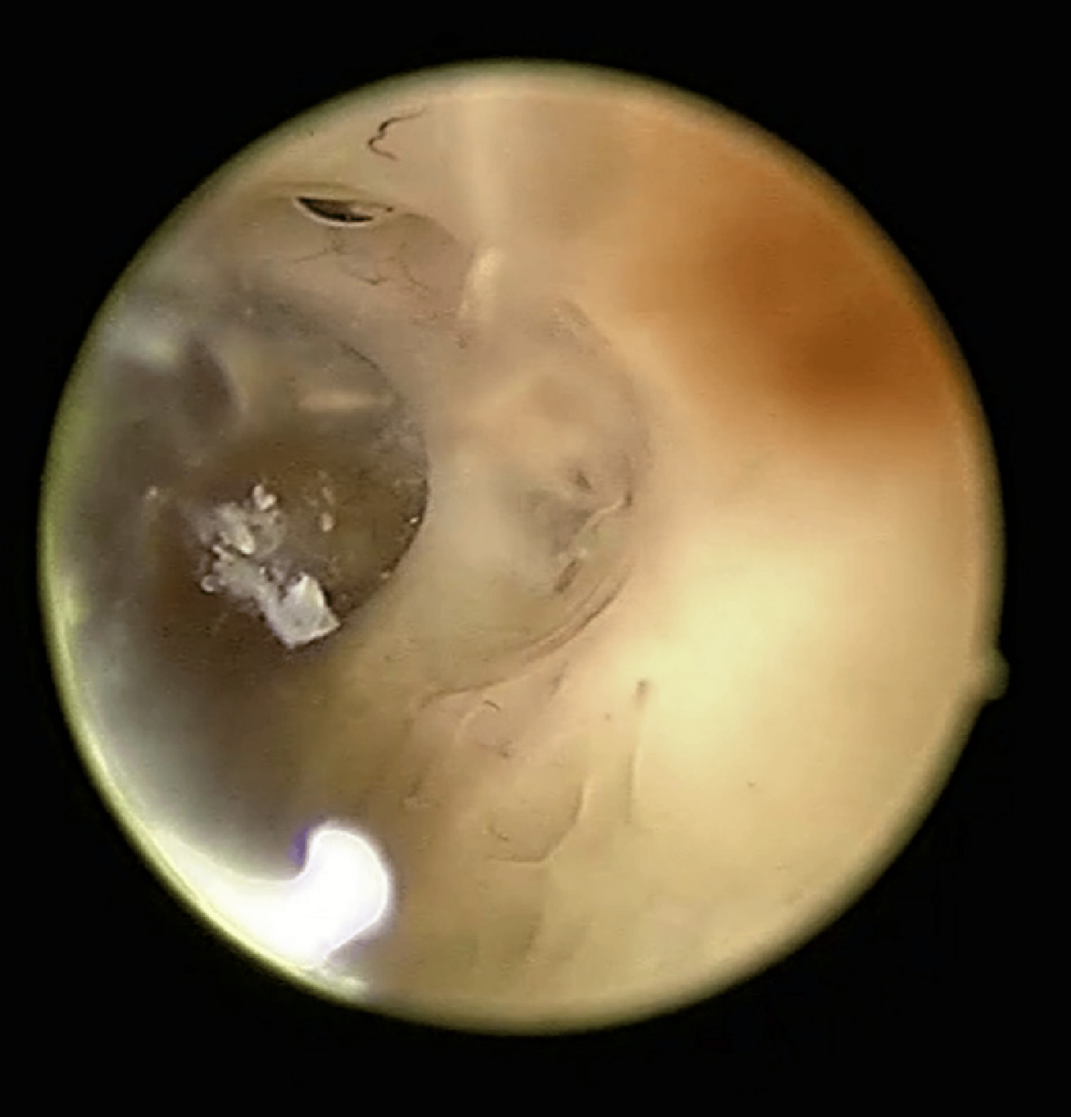
Left lateral uterine wall Hysteroscopic view of the left lateral uterine wall illustrating focal intrauterine adhesions (IUA) traversing the cavity and irregular areas of increased vascularity consistent with EVD. The adhesions appear as pale fibrotic bands connecting opposite endometrial surfaces, interspersed with reddish, tortuous vascular segments. The co-occurrence of EVD with IUA suggests a possible regenerative or reparative vascular response within fibrotic zones of the endometrium. The image highlights the diagnostic utility of hysteroscopy in differentiating structural and vascular abnormalities.

A 45-year-old multiparous lady, P4L4A1, with three vaginal births and one cesarean section, and tubal ligation done. She presented with a one and a half year history of menstrual irregularity and prolonged heavy bleeding with nil intermenstrual or postcoital spotting or pelvic pain. She had a normal ultrasound and pelvic and blood parameters. She was subjected to multiple episodes of short-term exposure to progesterone in the last year to control her menorrhagia. She underwent hysteroscopy and endometrial biopsy. Hysteroscopy revealed subtle IUAs and abnormal vascular patterns seen all over the uterus and spanning adhesions. The endometrium was irregular with increased vascularity. The endometrial sample confirmed a secretory endometrial pattern (Figure 5). The Appendix (Video 1) presents the summary of the case series.

## Discussion

In this case series, we present three women with a hysteroscopic diagnosis of EVD in the context of distinct endometrial and uterine pathologies, including IUAs, fibroids, endometriosis, a history of HIFU, and isthmocele. Our observations underscore the diverse clinical backgrounds of EVD and offer novel insight into the topographical relationship of vascular dystrophy within the endometrial cavity, particularly the notable absence of EVD in areas of isthmocele, while it exists on IUAs. This series aims to expand current understanding of EVD's clinical spectrum and its occurrence in the context of diverse uterine and endometrial pathologies, along with exploring its potential hormonal and structural correlates.

Pathophysiological considerations

The endometrial lining is constantly subjected to dynamic cyclical changes by fluctuations in estrogen, progesterone, and other endocrine signals, which collectively regulate tissue proliferation, differentiation, and vascular remodelling [4,5]. Estrogen predominates in the proliferative phase, inducing endometrial expansion, while the post-ovulatory surge of progesterone ushers in the secretory phase, marked by glandular tortuosity, luminal secretions, and stromal oedema [6]. In our series, histopathological assessment confirmed that EVD was consistently associated with secretory endometrium, characterized by well-developed sub- and supranuclear vacuoles and increasing intraluminal secretions - findings supported by prior studies [2,6].

Sopelana et al. [2] previously postulated that EVD primarily represents a morphological variant of secretory glands - tortuous and engorged with retained blood - rather than a primary vascular disorder. However, the mechanism for RBC entrapment within glandular lumina is not fully elucidated. As in earlier reports of Sopelana et al., our use of advanced histochemical stains confirmed that these intraluminal structures are bona fide endometrial glands filled with periodic acid-Schiff (PAS)-positive mucopolysaccharides and glycoprotein A-positive blood cells, indicating that trauma or procedural manipulation, such as curettage, may facilitate the ingress of erythrocytes into glandular spaces. The underlying pathophysiology of EVD remains incompletely explained; however, available evidence supports the concept that EVD likely reflects an exaggerated variant of the secretory-phase endometrium rather than a true vascular malformation. Progesterone-driven stromal oedema, sub- and supranuclear vacuolation, and enhanced secretory gland morphology during the luteal phase may predispose to transient entrapment of erythrocytes within dilated glandular lumina. This glandular distension, together with mucin-rich secretions, can mimic vascular ectasia under hysteroscopic visualization. In addition, local angiogenic modulators such as vascular endothelial growth factor (VEGF), matrix metalloproteinases (MMPs), and stromal cytokines - which are known to be dysregulated in endometriosis, fibroids, or chronic inflammation - may further contribute to altered microvascular remodelling in susceptible tissue environments. The coexistence of EVD within areas of IUA in our series suggests that focal regenerative responses within fibrotic zones might support selective glandular recovery, whereas the consistent absence of EVD atop isthmocele defects highlights that local tissue biomechanics, pressure gradients, or altered vascular perfusion may influence topographical expression of EVD within the uterine cavity.

The second case of our series, involving coexistent leiomyoma and endometriosis, supports the hypothesis that EVD may be associated with broader uterine pathologies and not solely dependent on the hormonal milieu.

Clinical correlates and associations

A key observation from our series is the consistent association of EVD with other uterine abnormalities: isthmocele, fibroid uterus, endometriosis, and IUAs. The exclusive sparing of EVD changes in the isthmocele zone, as seen in the first case of our case series, echoes findings from recent case reports, reinforcing the proposition that local tissue factors or altered microenvironments within isthmocele cavities may preclude the development of EVD features [3].

In the second case of our series with a fibroid uterus, endometriosis could have an intricate interplay of mechanical, hormonal, and angiogenic factors that could provide a conducive milieu for aberrant vascular remodelling, thereby facilitating EVD manifestation [7-9]. Both endometriosis and adenomyosis are associated with perturbed endometrial vascular architecture mediated by dysregulated angiogenic factors, such as VEGF, which in turn modulate stromal and glandular interactions [8,10]. The additional history of prior HIFU remains a novel possible association.

The coexistence of EVD with IUAs is another notable finding. IUAs typically result from trauma- or procedure-induced endometrial damage, triggering fibrous tissue deposition and impaired vascularization [11,12]. However, our demonstration of EVD patterns within areas of adhesion suggests that glandular regeneration may occur even within fibrotic zones, possibly due to focal reconstitution of endometrial function or microvasculature. The downregulation of VEGF in adhesions and its role in modulating stromal fibrosis may partially explain the patchy nature of EVD distribution in IUAs [12].

Diagnostic considerations

The diagnosis of EVD is primarily hysteroscopic, based on identification of dilated, tortuous, and occasionally thrombosed vascular structures in the endometrial lining [1-3]. However, differentiation from other vascular abnormalities remains a significant challenge. Enhanced myometrial vascularity (EMV), uterine arteriovenous malformations (AVMs), and retained products of conception may all present with abnormal endometrial or sub-endometrial vasculature but differ in their clinical implications and management [13,14]. The lack of pulsation in hysteroscopic vessels and the glandular, rather than vascular, nature of histological findings in EVD are important distinguishing features [1,2,15]. The diagnosis of EVD remains primarily hysteroscopic and requires careful differentiation from other vascular-appearing intrauterine abnormalities such as sub-endometrial arteriovenous malformations, retained products of conception, or hypervascular endometrial polypoid change. Hallmark features on hysteroscopy include multiple non-pulsatile, bluish, serpiginous channels diffusely distributed over the endometrial surface, often without active bleeding. However, hysteroscopy alone is insufficient to confirm whether these structures are dilated vessels or distended secretory glands; therefore, histopathology and adjunctive special stains (e.g., PAS, Alcian blue) are essential to demonstrate intraluminal mucin, confirming true glandular nature rather than vascular lumina. This diagnostic distinction is clinically relevant, as misclassification in favour of vascular pathology could prompt unnecessary embolisation or other invasive treatment. A structured diagnostic algorithm incorporating clinical context, phase of endometrial cycle, imaging correlates, hormonal exposure history, and targeted histopathology may improve recognition, avoid over-treatment, and increase awareness of this benign and likely self-limited entity.

Advanced histochemical staining remains indispensable in confirming that the observed vascular channels are, in fact, dilated glands containing erythrocytes and not malformed blood vessels [2]. This distinction is clinically relevant as it directly influences prognostic assessment and treatment decisions, averting unnecessary invasive interventions aimed at “vascular” pathology.

Implications and future directions

The benign, often self-limiting nature of EVD is underscored in our series by the symptomatic improvement of the first case following simple endometrial curettage, without the need for surgical correction of coexistent isthmocele. These findings support a conservative approach in the absence of other indications and highlight the need for further prospective studies to ascertain the natural history and clinical significance of EVD, especially in association with concurrent uterine pathologies.

Our observations also raise important mechanistic questions:

(1) Is EVD a primary histological entity or a secondary phenomenon arising from tissue response to hormonal, mechanical, or procedural stimuli?

(2) What are the local factors that result in the absence of EVD within areas such as isthmocele?

(3) Can EVD serve as a marker of endometrial pathology in broader gynecological practice?

Further research employing larger cohorts, precise hormonal profiling, and advanced endometrial imaging and molecular analysis is warranted to elucidate the underlying mechanisms and clinical significance of this enigmatic finding.

Study strengths and limitations

This series is strengthened by the detailed hysteroscopic, histopathological, and clinical correlations provided, including the use of advanced staining techniques to clarify the nature of EVD. However, as a small observational case series, our findings are hypothesis-generating and require validation in larger, prospective datasets to delineate causality and clinical implications.

## Conclusions

This case series expands the clinical spectrum of EVD, demonstrating its occurrence across a variety of gynecological backgrounds, irrespective of parity or exogenous progesterone exposure. Our findings underscore the predominantly benign and likely self-limited nature of EVD, consistent with prior observations. Notably, we observed site-specific EVD morphology, with the condition manifesting within certain pathological endometrial contexts such as intrauterine adhesions, yet notably absent within isthmocele regions. These spatial patterns suggest that local tissue factors and underlying uterine pathologies may influence the development and distribution of EVD. Moreover, the presence of EVD in patients with complex clinical histories, including endometriosis, leiomyomas, and prior uterine interventions, highlights the potential contribution of both hormonal and structural factors to its pathogenesis, regardless of progesterone exposure.

Given the rarity of reported cases and the limited understanding of its clinical implications, ongoing documentation and systematic investigation are warranted to clarify the long-term outcomes and management strategies associated with EVD. We encourage further case reporting and larger collaborative studies to determine whether EVD has any prognostic significance or requires intervention and to refine diagnostic criteria for distinguishing it from other endometrial vascular abnormalities.

## Appendices

By contributing these additional cases, supplemented with detailed hysteroscopic high-definition quality video visualization and pictures, we aim to enhance recognition and understanding of EVD among clinicians and pathologists, ultimately facilitating more informed patient care and research into this underrecognized endometrial entity (Video 1).
